# Customizable Nichrome Wire Heaters for Molecular Diagnostic Applications

**DOI:** 10.3390/bios14030152

**Published:** 2024-03-20

**Authors:** Juhee Lim, Won Han, Le Tran Huy Thang, Yong Wook Lee, Joong Ho Shin

**Affiliations:** 1 Industry 4.0 Convergence Bionics Engineering, Pukyong National University, Busan 48513, Republic of Korea; ij8709@pukyong.ac.kr (J.L.); hanwon0427@pukyong.ac.kr (W.H.); lethang@pknu.ac.kr (L.T.H.T.); yongwook@pknu.ac.kr (Y.W.L.); 2 School of Electrical Engineering, Pukyong National University, Busan 48513, Republic of Korea; 3 Major of Biomedical Engineering, Division of Smart Healthcare, College of Information Technology and Convergence, Pukyong National University, Busan 48513, Republic of Korea

**Keywords:** nichrome wire, heater patterning, customizable heater, pathogen detection

## Abstract

Accurate sample heating is vital for nucleic acid extraction and amplification, requiring a sophisticated thermal cycling process in nucleic acid detection. Traditional molecular detection systems with heating capability are bulky, expensive, and primarily designed for lab settings. Consequently, their use is limited where lab systems are unavailable. This study introduces a technique for performing the heating process required in molecular diagnostics applicable for point-of-care testing (POCT), by presenting a method for crafting customized heaters using freely patterned nichrome (NiCr) wire. This technique, fabricating heaters by arranging protrusions on a carbon black-polydimethylsiloxane (PDMS) cast and patterning NiCr wire, utilizes cost-effective materials and is not constrained by shape, thereby enabling customized fabrication in both two-dimensional (2D) and three-dimensional (3D). To illustrate its versatility and practicality, a 2D heater with three temperature zones was developed for a portable device capable of automatic thermocycling for polymerase chain reaction (PCR) to detect *Escherichia coli* (*E. coli*) O157:H7 pathogen DNA. Furthermore, the detection of the same pathogen was demonstrated using a customized 3D heater surrounding a microtube for loop-mediated isothermal amplification (LAMP). Successful DNA amplification using the proposed heater suggests that the heating technique introduced in this study can be effectively applied to POCT.

## 1. Introduction

In vitro diagnostics are techniques utilized for genetic and biomarker tests, establishing the standard for detecting various infectious diseases. The diagnostic methods can be largely divided into two: immunoassays and molecular diagnostics. An example of immunoassay is lateral flow immunoassay (LFIA), leveraging antigen–antibody reactions; and examples of some of the most widely used molecular diagnostics are polymerase chain reaction (PCR) and loop-mediated isothermal amplification (LAMP), operating based on nucleic acid amplification tests (NAATs). LFIA, employed in point-of-care testing (POCT), serves to confirm the presence or absence of a target by utilizing the interaction between the antigen in the collected sample and the immobilized antibody on the strip. Despite its simplicity and rapidity, LFIA has a notable drawback—a high limit of detection (LOD) influenced by the quantity of antigen in the collected sample. The LOD, representing the threshold at which a target can be identified in a specific sample, serves as a crucial metric for evaluating device performance. This means that devices with lower LOD demonstrate higher sensitivity. In contrast to LFIA, NAATs, a laboratory-based technology requiring specialized equipment, boast a lower LOD due to the exponential amplification of target nucleic acid (NA) such as DNA or RNA [1,2]. Despite the need for a preprocessing step involving the separation, purification, and extraction of NA from collected samples, NAATs offer this distinct advantage of high sensitivity. The NA extraction process involves combining the sample with lysis buffer and enzymes, followed by incubation at temperatures of 55 °C, 70 °C, or 90 °C [3]. Omitting the heating process during DNA extraction results in a significant reduction in the concentration of extracted nucleic acids. This heating step is equally pivotal during the amplification of extracted DNA. In PCR, the amplification process occurs through the separation and replication of double-helix strands via a thermal cycling process [4]. In contrast, LAMP achieves DNA amplification by forming a loop structure under isothermal conditions [5,6]. Thus, heating the sample becomes an indispensable element in DNA extraction and NAATs.

Various attempts have been made to apply NAATs, which offer the advantage of low LOD, to the POCT environment [7,8,9,10]. Microfluidic chip-based PCR has the advantage of requiring only a small sample volume for PCR reactions. However, complex fabrication steps are necessary for making microfluidic chips, and these chips are generally intended to be disposable. Moreover, integrating NAATs into POCT, typically conducted in laboratory settings, necessitates devices capable of replacing bulky heating instruments like thermal cyclers. In response, numerous research groups have developed devices utilizing various types of heaters to facilitate the application of NAATs in POCT. Among them, a method utilizing the thermal insulation of a tumbler to maintain the boiling water temperature without requiring additional electricity during sample heating has been proposed for easy use in the POCT environment [11,12]. However, it requires refilling of boiling water for reuse. Another non-electric heating technology involves heating water through a chemical reaction [13,14,15,16]. This technology is cost-effective and exhibits a rapid reaction rate, but it is not safe for unskilled users. Additionally, the use of disposable chemicals raises concerns about environmental pollution due to chemical waste. In addition, when providing heat non-electrically, there is a challenge of short heating duration and difficulty in sustaining the heat. Therefore, for stable and reliable heating, the use of electricity is typically involved.

Among previous studies that have conducted NAATs in POCT environments using electrically powered heaters, thermoelectric modules stand out as a widely preferred option. They are commonly employed in commercial thermocyclers, offering high ramp rates and the versatility to function as both heaters and coolers, depending on the polarity of the power supply [17,18,19,20,21,22,23]. Despite these advantages, Peltier heaters have limited energy efficiency and heat flux. They also need a large heat sink and turbofan to dissipate the generated heat, which limits their portability and their application in POCT [24]. Other groups employed cartridge heaters since they are readily available with different standards and dimensions and are cheaper than thin-film heaters and Peltier heaters [8,25,26,27,28,29]. However, they usually require a cooler for thermocycling applications [28]. Other researchers experimented with microwave heaters and demonstrated an ultra-fast ramping rate, up to 65 °C/s [30,31,32]. However, they are inefficient, and is difficult to control the temperature [33]. Some groups have utilized lasers as heaters since they allow non-contact and local heating by having embedded conductive material such as carbon black or graphite inside the microfluidic chip [34], demonstrating low energy consumption and high ramping rate [35]. Nevertheless, high-power lasers and peripheral hardware to control the laser’s power are expensive. Laser heaters also require precise alignment between the lasers and objects to work properly. Another approach is to use positive temperature coefficient (PTC) heaters. Since they have an open-loop control system, PTC heaters do not require a complicated temperature control system and are less susceptible to overheating [36,37,38,39,40]. However, they typically need a heat sink or fan to dissipate the heat. Thin film heaters are a widely used technology owing to their low power consumption and high application potential, and they are also commonly used in the fields of POCT and isothermal amplification [41,42,43,44,45,46,47]. They are small, lightweight, and flexible, with high watt density and low thermal mass, capable of running on battery [41]. This heater also has the advantage of avoiding the use of bulky instruments [45]. Nevertheless, thin-film heaters, particularly polyimide heaters, exhibit a constrained temperature range, are susceptible to edged bending, and may fail if folded abruptly [48].

Many of the abovementioned heaters, especially commercial heaters generally have a fixed shape, limiting the customization of heaters to desired two-dimensional (2D) and three-dimensional (3D) forms. Such customization is needed to facilitate the research and development of next-generation NAATs, and can also contribute to the miniaturization and implementation of NAATs for POCT applications, which has proven to be important during the COVID-19 pandemic. Recent research shows efforts to enable the custom fabrication of heaters. Techniques involving 3D printing using graphene materials have been explored to produce heaters in desired shapes [49,50,51]. However, graphene materials are generally expensive, and the fabrication of heaters necessitates the use of a 3D printer each time. Consequently, there is a limitation in terms of high fabrication costs and dependency on the performance of the 3D printer when producing heaters. In this study, we propose a simple technique of patterning nichrome (NiCr) wire to create customized heaters. The technique proposed in this study distinguishes itself by utilizing low-cost and readily available materials. The NiCr wire used for custom heater fabrication has been commonly employed in many household devices such as hairdryers and toasters but has not been applied in molecular diagnostics research. We patterned NiCr wire onto a cast of carbon-black embedded polydimethylsiloxane (PDMS) with regularly spaced protrusions to fabricate user-customized heaters. The presented heater is small, cost-effective, and lightweight, offering superior portability compared to Peltier heaters, cartridge heaters, microwave heaters, and PTC heaters. Additionally, our customized NiCr heater, as compared to laser-based heaters, offers ease of use and the ability to shape not only complex patterns in 2D but also 3D structures without compromising heating performance when compared to thin film heaters. We demonstrate the amplification of the DNA of *Escherichia coli* (*E. coli*) O157:H7 pathogen by utilizing a customized heater fabricated by the proposed technique. A device capable of conducting a thermal cycling process was fabricated using a 2D patterning, and was used to perform PCR. Additionally, a 3D heater that wraps around a tube was fabricated and applied to perform LAMP. As a result of the experiment, the presence or absence of the target DNA could be confirmed through gel electrophoresis and colorimetric reactions, proving that the heater fabrication technique presented in this study is suitable for use in molecular diagnostics.

## 2. Materials and Methods

### 2.1. Reagents and Materials

Ecoflex base and curing agent were purchased from Smooth-On (Ecoflex 0030, Macungie, PA, USA). PDMS base and curing agent were purchased from Dow (SYLGARD 184, Macungie, MI, USA). Activated carbon black powder (Carbon black) was purchased from Sigma-Aldrich (702102-5G, St. Louis, MO, USA). For surface coating, trichloro (1H, 1H, 2H, 2H-perfluorooctyl)silane (PFOTS silane) was purchased from Sigma-Aldrich (448931, St. Louis, MO, USA). The 42-gauge NiCr wire, consisting of 80% nickel and 20% chromium, used for patterning, was purchased from NK (B08P5KH4YJ, Nanjing, China). The simulation was conducted using COMSOL Multiphysics software (v5.6, Burlington, MA, USA). PureLink Genomic DNA Mini Kit was purchased from Thermo Fisher Scientific (K182000, Waltham, MA, USA). All primers used for DNA amplification were synthesized by Bioneer (Daejeon, Korea). WarmStart^®^ Colorimetric LAMP 2X Master Mix (DNA and RNA) was purchased from New England BioLabs (M1800S, Ipswich, MA, USA). amfiSure PCR Master Mix(2X) (P0311-010), gel loading dye (S1001-025) DNA ladder (V1002-100) was purchased from GenDEPOT (Katy, TX, USA).

### 2.2. NiCr Wire Patterning Heater Fabrication

**Figure 1:**
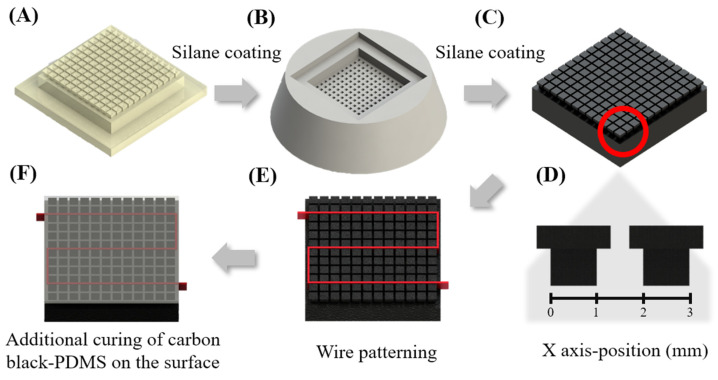
Scheme of heater fabrication method. (**A**) The positive resin mold was created through 3D printing. (**B**) A negative Ecoflex mold was then modeled after the positive resin mold. (**C**) The cast was generated by curing Carbon Black-PDMS in the negative Ecoflex mold. (**D**) Zoomed-in side view of the protrusion (indicated by the red circle in (**C**) showing T-shaped structures. (**E**) Nichrome wire (indicated by the red line) is weaved through the protrusions in a desirable path. (**F**) the gap between the T-shaped protrusions was filled with Carbon Black-PDMS to secure the wire, and the material was subsequently hardened.

A positive resin mold (Figure 1A) identical to the protrusion pattern of the heater was produced using a 3D printer (Form 3, Form Lab, Somerville, MA, USA) and resin (Standard resin, Form Lab, Somerville, MA, USA). To treat the surface of the mold, chemical vapor deposition (CVD) was performed using PFOTS silane. Afterward, the negative Ecoflex mold is fabricated by curing the Ecoflex base and curing reagent at a 1:1 ratio on the treated resin mold as shown in Figure 1B. For the carbon black PDMS mixture, we mixed the PDMS base and curing agent in a 10:1 ratio, incorporating 1% weight/weight (*w*/*w*) carbon black into the mixture. After degassing the carbon black PDMS mixture for one hour, we cured it in a negative Ecoflex mold in an oven at 80 °C for 30 min. Through this process, the carbon black-PDMS cast shown in Figure 1C is fabricated. The surface of the carbon black-PDMS cast is arranged with T-shaped protrusions as shown in Figure 1D To pattern a 42-gauge NiCr wire onto the designed shape, we utilized the protrusions of the fabricated carbon black PDMS cast as shown in Figure 1E Subsequently, an additional curing process was conducted by filling the space between the protrusions with carbon black-PDMS to secure the wires as shown in Figure 1F. The thickness of the heater fabricated through this process is 4 mm, and the NiCr wire is located 1.3 mm deep from the heater surface. The surface temperature of the heater was assessed using an infrared (IR) camera from Teledyne FILR (FLIR one Pro, Wilsonville, OR, USA).

### 2.3. Bacteria Preparation

*E. coli* O157:H7 (ATCC 35150) was cultured in 5 mL of Luria Bertani (LB) broth for 18 h in a shaking incubator at 37 °C and 200 rpm. LB broth was prepared by combining peptone, sodium chloride, and yeast extract in a ratio of 5:5:2.5 (grams) in 500 mL of deionized (DI) water. Identification of *E. coli* O157:H7 was carried out using the colony counting method, and optical density (OD) was measured at a wavelength of 600 nm. The solution was subsequently diluted to a concentration of 10^8^ based on the measurement of OD, and DNA extraction was performed following the PureLink Genomic DNA Mini Kit instructions.

### 2.4. PCR Solution Preparation

The PCR sample, with a total volume of 25 μL, was prepared by adding 12.5 μL of master mix and 0.625 μL each of the forward and reverse primers to 9.25 μL of DI water. Subsequently, 2 μL of the extracted DNA sample was added to complete the preparation.

### 2.5. 2D Heater Patterning for the Fabrication of a Rotary-Type Thermocycler

The Rotary PCR device is designed to perform thermocycling by sequentially rotating a chamber containing PCR solution on a circular heater that has three zones with different temperatures. Utilizing SolidWorks software 2020 from Dassault Systèmes (Vélizy-Villacoublay, France), the rotating body, which integrates the holder and chamber of the three-layer device, was conceptualized. The holder comprises a circuit layer, a step motor fixing layer, and a heater layer. The circuit layer and step motor fixation layer were 3D printed using PLA filament, while the upper heater layer was constructed with high-temperature resin (high temp resin, Form Lab, USA) possessing a high heat deflection temperature.

### 2.6. PCR Chamber Fabrication

The PCR chamber was crafted using a polypropylene (PP) bag. To treat the surface of the PP bag, the same PFOTS silane coating process employed in the heater fabrication was applied. Subsequently, the surface-treated bag was hermetically sealed using a sealing machine to prevent evaporation. Each PCR chamber accommodates 25 μL of solution, with dimensions approximately measuring 1 cm in width and 1 cm in length.

### 2.7. LAMP Solution Preparation

The primers utilized in this study were produced following the same conditions as those in the preceding research. For the preparation of the LAMP solution sample, 25 μL of WarmStart^®^ Colorimetric LAMP 2X Master Mix and 5 μL of primers were combined with 16 μL of deionized (DI) water, followed by the addition of 4 μL of extracted DNA. The total volume of LAMP solution samples was adjusted to 50 μL and all samples were prepared in 1.5 mL tubes.

### 2.8. 3D Heater Patterning for the Fabrication of a Tube-Shaped Heater

To fabricate a 3D heater enveloping a 1.5 mL microcentrifuge tube, the identical procedure employed in fabricating a flat heater was implemented. Initially, a resin mold with a raised pattern was created using 3D printing, and subsequently, an Ecoflex mold was produced based on this design. NiCr wire was patterned on a mold formed by curing a carbon black PDMS mixture in an Ecoflex mold. To fix the pattern of the wire, a structure made of resin was used to fix the mold in which the wire was patterned, and then carbon black PDMS was additionally poured and cured. For the measurement of the temperature of the water that is heated by the tube-shaped heater, blue dye was added to the water because accurately measuring the temperature of a transparent medium using an IR camera presents difficulties due to light refraction [52]. It was verified that a temperature stability of ±2 °C was consistently maintained for over 2 h at the designated target temperature of 65 °C.

## 3. Results and Discussions

### 3.1. The Selection of Material for Heater Fabrication

**Figure 2:**
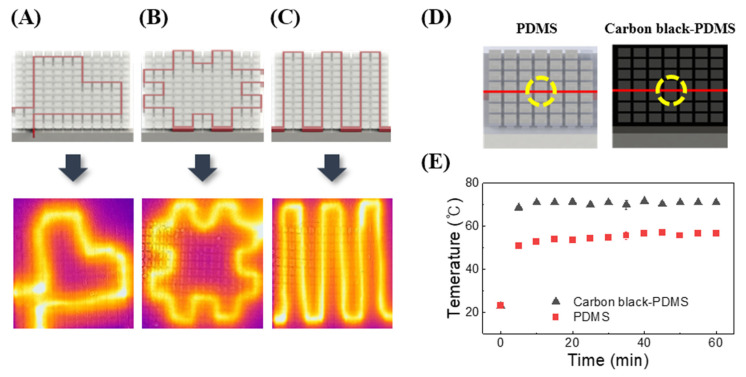
Heater customization by PDMS wire patterning. Schematic representation of the PDMS cast (**top**) for wire (indicated by red line) patterning, and the IR camera images (**bottom**) showing the heater patterned in the form a (**A**) a heart, (**B**) a hashtag, and (**C**) a wave. (**D**) Schematic representation showing a heater designed to compare the thermal conductivity with and without 1% carbon black added to PDMS (yellow circle indicates the region of interest for the temperature measurement using an IR camera). (**E**) Graph showing the measured temperature over time at 5 while applying 3 V voltage to each heater.

In this study, PDMS was chosen as the material for fabricating custom heaters due to its simplicity in molding and ease of fabrication. Following the fabrication, NiCr wires were patterned onto the PDMS cast to create a custom heater. The temperature changes on the heater surface were then monitored using an IR camera. The monitoring results revealed that the heater effectively heated in a patterned form on the PDMS cast as shown in Figure 2A–C. However, a drawback was identified, indicating the need for a high voltage due to the low thermal conductivity of PDMS. PDMS, a polymer with silicon as its main component, exhibits a low thermal conductivity in the range of 0.1 to 0.3 W/mK [53,54,55], like other polymers. Recognizing the limitation of using pure PDMS for heater fabrication due to its low thermal conductivity, carbon black was employed to enhance thermal conductivity. Carbon black, commonly used as an additive in polymer composites, serves the role of improving the thermal conductivity of the composite by forming conductive paths through particle-to-particle contact [56]. Referring to previous studies [5], carbon black was added to PDMS at a concentration of 1% (weight/weight) during the cast fabrication process. Two heaters were fabricated under identical conditions, excluding the presence or absence of carbon black, to compare the thermal conductivity of heaters with and without carbon black as shown in Figure 2D. The NiCr wires were linearly affixed to structures with protrusions (indicated by straight red lines in Figure 2D), without any specific pattern, and the overall size of the heaters was designed to be 15 mm × 15 mm × 4 mm (width × depth × height). The experimental results confirmed that the heater’s surface, utilizing the carbon black-added cast, exhibited a higher temperature under identical voltage conditions as shown in Figure 2E. This phenomenon can be attributed to the elevated thermal conductivity of carbon black, validating the potential for further enhancing energy efficiency through the fabrication of heaters with carbon black embedded PDMS, as evidenced by the experimental results. Thus, for the rest of the study, 1% carbon black was mixed with PDMS for the fabrication of heaters.

### 3.2. Thermal Profiling

#### 3.2.1. Simulation for the Determination of the Wire Spacing

**Figure 3:**
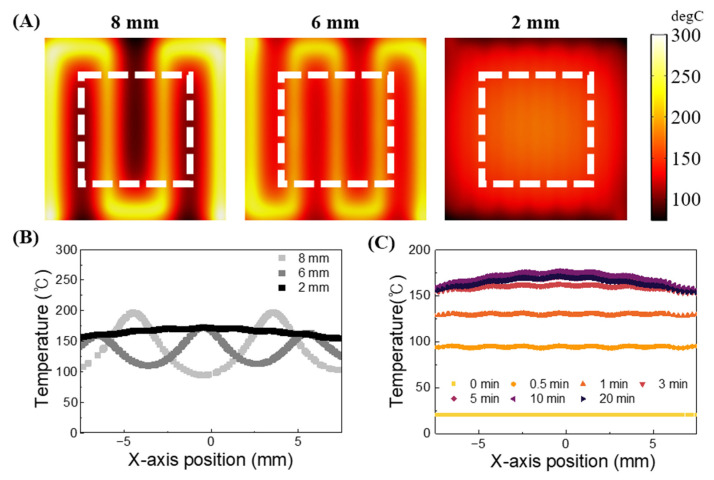
Simulation results of heater temperature profile according to wire spacing distances. (**A**) Images and (**B**) graph depicting the temperature distribution on the heater surface based on the difference in wire spacing. The images display the temperatures of each heater patterned with wires at intervals of 8 mm, 6 mm, and 2 mm after applying a 10 V voltage, measured 20 min later. The graph represents the temperature distribution in the area within the white frame of the image. (**C**) The temperature change on the heater surface over time is depicted when applying a 10 V voltage to the heater with wires patterned at 2 mm intervals.

For molecular diagnostics, maintaining a uniform heating of samples at the correct temperature is crucial. Thus, to apply the heater fabrication technique presented in this study to molecular diagnosis, it is essential to ensure the uniformity of the surface temperature of the fabricated heater. The spacing between wires can influence the heater’s surface temperature gradient. The suggested heater is designed with protrusions for convenient patterning and securing of wires, and the patterning spacing of the wires can be controlled by adjusting the size of the protrusions. Consequently, we aimed to compare the surface temperature gradients of heaters with wire spacings of 2 mm, 6 mm, and 8 mm, respectively, through simulation. The simulation was performed using a 25 mm × 25 mm × 4 mm heater. To reduce the effect of heat loss on the surrounding environment and ensure effective sample heating, an area of ±7.5 mm horizontally and ±7.5 mm vertically from the center of the heater was designated as the sample heating region (indicated by white dotted box), upon which the analysis was conducted, as shown in the white squares in Figure 3A. The simulation results confirmed that the temperature deviation decreases as the wire spacing becomes narrower as shown in Figure 3B. The heater with wires patterned at 2 mm intervals exhibited a more uniform temperature compared to the heaters with wires patterned at 6 mm and 8 mm intervals. Based on these findings, in this study, protrusions were fabricated to ensure a 2 mm gap between wires, aiming to achieve a uniformly distributed temperature on the heater surface. Additionally, simulations were conducted to verify the stability of the surface temperature of the fabricated heater over time. The simulation results indicated that the temperature of the heater surface remained within a certain range after five minutes of applying voltage to the wire as shown in Figure 3C. Based on the simulation results, we verified the wire spacing of 2 mm was enough to maintain a uniform temperature and confirmed that the temperature of the fabricated heater converges over time. This allowed us to assess the performance of the heater proposed in this study.

#### 3.2.2. Observation of the Heater Surface Temperature with Respect to the Applied Voltage

**Figure 4:**
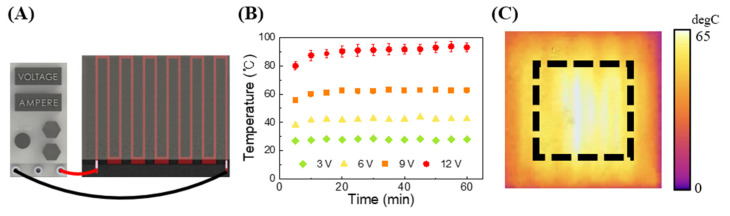
(**A**) Scheme of the experimental setup. The power supply provides voltage to the wire-patterned heater. (**B**) Temperature distribution based on voltage in a heater fabricated with patterns at 2 mm intervals. The experimental heater size was 25 mm × 25 mm (width × length). Temperature changes were measured at three different points within the black frame in the (**C**) IR camera image.

The presented wire-based custom heater operates using a DC voltage as the power source as shown in Figure 4A. When an electrical current passes through the heater, the high resistance of the NiCr wire leads to the conversion of electrical energy into heat energy, in other words, Joule heating occurs. Increasing the voltage applied to the NiCr wire results in a greater temperature increase. Based on this principle, we gradually increased the voltage applied to the proposed customized heater to observe the temperature changes on the heater surface. After applying voltage to the wire, we monitored the temperature changes on the heater surface for one hour to conduct the experiment shown in Figure 4B. The temperature measurement using the IR camera is depicted in Figure 4C, and as in the previous simulations, we measured the temperature within the designated sample heating area indicated by the black rectangle (±7.5 mm horizontally and ±7.5 mm vertically). The measurement results confirmed that the surface temperature of the heater increased with higher applied voltage. Consequently, it was confirmed that by adjusting the voltage, it is possible to set and achieve the target temperature required for experiments when using the suggested heater. Furthermore, the temperature of the heater surface was 93 ± 4 °C when a voltage of 12 V was applied, falling within the appropriate temperature range for the denaturation stage of PCR, which is the highest temperature required for the NA amplification. Therefore, it was confirmed that the heater fabricated using the technique presented in this study can meet the temperature required to perform PCR.

### 3.3. Applying NAATs for on-Site Diagnosis Pathogenic Bacteria

#### 3.3.1. Fabricating Thermal Cycling Device and Performing PCR

**Figure 5:**
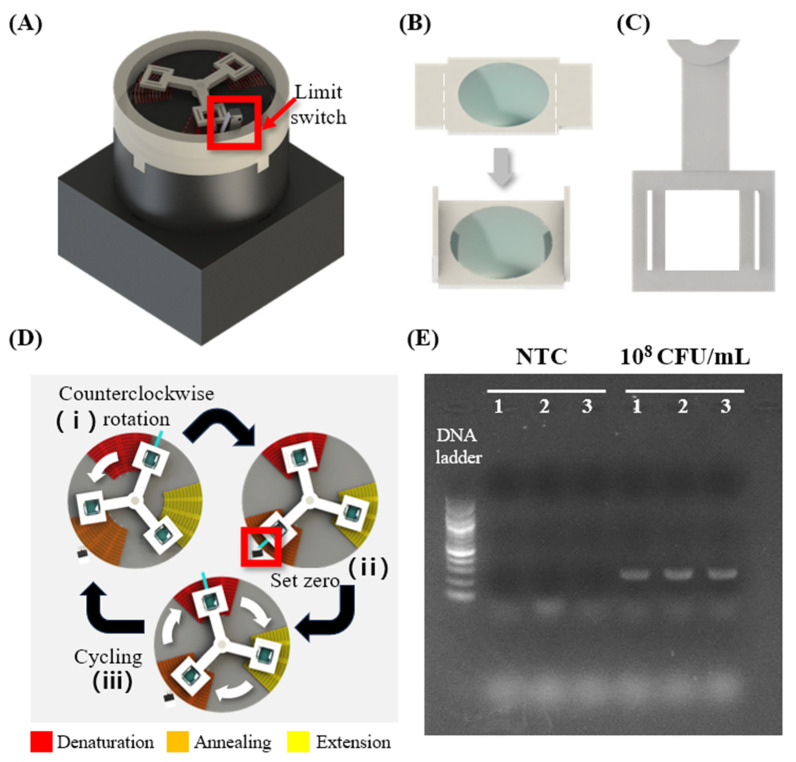
(**A**) Appearance of the PCR device. (**B**) Chamber containing PCR samples. Both wings can be folded and assembled into the hole of the (**C**) rotating body connected to the motor. (**D**) Schematic diagram showing the thermal circulation process using three different temperature zones: Denaturation (95 °C), annealing (60 °C), and extension (72 °C). When the rotating body is connected to the motor and power is applied, the (i) motor rotates counterclockwise. (ii) When the rotating body pushes the limit switch, the limit switch is activated. (iii) When the limit switch, functioning as the origin, is activated, the motor undergoes a process of rotating 120 degrees clockwise followed by a 1-min pause, and this sequence is repeated three times (Red is the denaturation section, orange is the annealing section, and yellow is the extension section). Through this rotation, the chamber can undergo a thermal cycling process. After one thermal cycling, the motor will rotate counterclockwise. This rotation can reactivate the limit switch, thereby allowing the thermal cycle to continue. (**E**) Gel electrophoresis image. Numbers of the lanes indicate the repeats of the experiments, which were performed in triplicate.

The proposed customized heater fabrication technique was employed to develop a device suitable for use in the PCR. The device, as depicted in Figure 5A, can carry out the thermal cycling process required for PCR by rotating the chamber containing the sample. Sequentially placing the reaction tube on heating zones with different temperatures rather than changing or cycling a single heat block’s temperature is popular in the field of microfluidics and POCT research [57,58,59,60]. It effectively reduces the time required to finish PCR by eliminating the time required for ramping up and down the heat block. For the fabrication of the PCR chamber, PP bags commonly used for packaging were utilized. To modify the surface properties of the PP material bags, a coating process involving PFOTS silane was implemented. This process serves to inert the surface of the bags, preventing unwanted interactions between the sample and the material. The chamber was fabricated by sealing it using a sealing machine and then attaching a PP film as shown in Figure 5B. The PP film is designed with a rectangular structure matching the chamber size, featuring wing structures on both sides. The design allows for folding both wings of the film and inserting them into the holes of the rotating body as shown in Figure 5C. The rotating body combined with the chamber is connected to a motor and rotates. To achieve good thermal contact between the PCR chamber and the heater surface while also allowing a smooth rotation of the chamber, we created a small gap of 1 mm or less between the heater surface and the chamber, filled the gap with thermal paste to facilitate conductive heat transfer to the chamber. Additionally, by applying thermal paste along the chamber’s path of movement, we reduced friction between the heater surface and the chamber, ensuring smooth chamber rotation. The initial version of the device was based on a programmed rotation of a step motor to drive the rotor. However, step motors available on the market have a problem: they do not stop at a consistent position each time when repeating a series of alternating rotations. To address this issue, a limit switch was employed as shown in Figure 5A red box. The limit switch serves as the origin, and the motor rotates 120 degrees clockwise and stops, repeating the process three times, and then rotating counterclockwise to activate the limit switch. Through the repetition of this cycle, the rotating body consistently returns to the exact origin as shown in Figure 5D. This allows the rotor to heat the chamber containing the PCR sample at the correct location to perform thermal cycling. The PCR process demands three temperatures: denaturation (95 °C), annealing (60 °C), and extension (72 °C). In this study, the temperature criteria were established based on the sample inside the chamber reaching the target temperature required for the PCR process, as observed through an IR camera. At this point, the temperature of the heater surface was 113 °C for the denaturation zone, 55 °C for the annealing zone, and 80 °C for the extension zone. The chamber is designed to dwell for 1 min at each temperature area, taking approximately 3 min per cycle. In this study, an experiment was conducted to amplify E. coli O157:H7 DNA using the device. Three chambers containing non-template control (NTC) without target DNA, and three chambers containing DNA at a concentration of 10^8^ CFU/mL were subjected to 30 cycles of thermocycling, and then gel electrophoresis was performed to confirm the results as shown in Figure 5E. In gel electrophoresis experiments, DNA fragments within an agarose gel migrate under the influence of an electric field [61]. Depending on the size of the DNA, they travel different distances within the agarose gel, allowing for the separation of DNA based on size. The migrated DNA fragments form bands at specific locations in the gel. For the NTC, no band was formed during electrophoresis due to the absence of DNA. Conversely, samples containing target DNA at a concentration of 10^8^ CFU/mL exhibited a visible target band after electrophoresis. These results confirm the successful execution of the PCR process through the proposed heater fabrication technique. The device presented here is designed with a focus on the on-site diagnostic application of customized heater fabricating technology, resulting in a relatively longer detection time compared to other PCR devices for POCT. However, previous research [58] has demonstrated that optimization of temperature conditions to enable annealing and extension simultaneously can shorten detection time, as opposed to heaters with three temperature zones [59]. By applying this approach to the device presented in this study and fabricating a heater with two temperature zones required for denaturation and annealing/extension, we can expect a reduction in detection time.

#### 3.3.2. Fabrication and Application of 3D Heater for Isothermal Amplification

**Figure 6:**
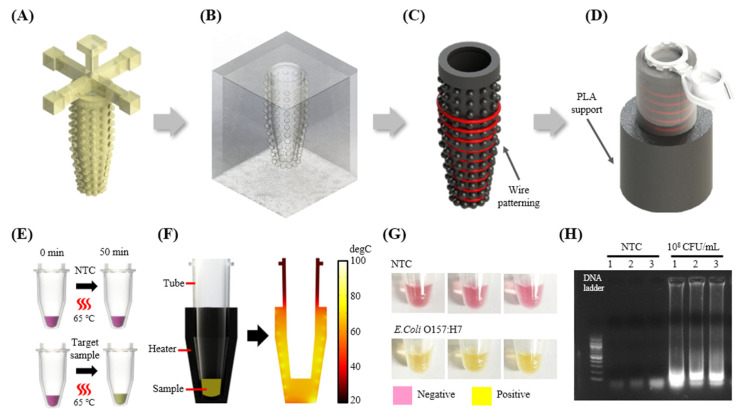
(**A**) Positive resin mold. (**B**) Negative Ecoflex mold fabricated based on the positive resin mold. (**C**) Patterning the NiCr wire (indicated by red line) by rotating it nine times on a Carbon black-PDMS cast, fabricated based on the Negative Ecoflex mold. (**D**) The microtube and the fabricated heater are combined with the PLA support to stably fix the heater. (**E**) Scheme of LAMP colorimetric reaction. (**F**) Simulation image showing the temperature distribution inside the sample when heating the sample using the fabricated heater. (**G**) LAMP colorimetric reaction result. (**H**) Gel electrophoresis image. Experiments were performed in triplicate.

The customized heater fabrication technique presented in this study extends beyond 2D shapes, allowing for the creation of 3D shapes as well. To demonstrate this, a compact heater was designed to heat the sample inside the microtube as shown in Figure 6A–D. Unlike 2D heaters, this 3D heater can effectively transfer heat to the tube by surrounding and providing good thermal contact over the curved surface of the tube, enabling conductive heating. Winding the wire around the tube-shaped structure allows for secure fixation, eliminating the issue observed in the fabrication of 2D heaters where the wire is not securely fixed and protrudes higher than the structure’s surface. Consequently, the design of the protrusion was simplified and crafted into a hemispherical shape. This hemispherical protrusion serves to control the spacing between wires during the wire patterning. With a heater designed to surround the microtube for isothermal amplification, the aim was to perform LAMP experiments displaying a colorimetric response as described in 6E. To verify whether the sample could be uniformly heated when using the fabricated heater, simulations were conducted as shown in Figure 6F. The simulation results confirmed that when a voltage of 5V was applied to a heater constructed by rotating wires nine times, it could uniformly heat the sample to the target temperature of 65 °C. To apply this 3D heater in isothermal amplification, we intended to amplify the same DNA used for PCR, specifically, *E. coli* O157 DNA. The heater temperature was adjusted to reach 65 °C, the required temperature for performing LAMP, and the reaction was carried out through one hour of heating. The experimental results showed that there were no visible color changes in the case of the NTCs, and during gel electrophoresis, no specific bands could be identified. This is attributed to the absence of target DNA in the samples. In contrast, for the sample with a concentration of 10^8^ CFU/mL of target DNA, a visible color change was observed, and a target band was evident during gel electrophoresis as shown in Figure 6G,H. Based on these results, the potential of the 3D compact heater proposed in this study for on-site diagnostics in the field has been confirmed.

Furthermore, if a system capable of controlling the temperature during the heating process required for NA amplification is introduced, we anticipate that by sensing and regulating the temperature of the heater to maintain a constant temperature, the stability and performance of the heater can be enhanced [62]. This, in turn, is expected to further improve the POCT suitability of the presented heater fabrication technology. While our paper does not specifically address this aspect in detail, it hints at potential avenues for future development.

## 4. Conclusions

In this study, a NiCr wire patterning-based heater fabrication technique is introduced to facilitate the required heating for molecular diagnosis in POCT. This heater fabrication technique has the advantage of easy customization using readily available materials. The surface of the cast, made of carbon black-PDMS material, features arranged protrusions to facilitate wire patterning. By adjusting the size of the protrusions, users can easily control the distance between the wires or control the shape and size of the heated area. The heater can regulate temperature by adjusting the voltage, and it was confirmed to operate stably at the target temperature. The presented heater demonstrated stability in maintaining a consistent temperature, even at the highest temperature used in PCR, namely 95 degrees, confirming its suitability for NA amplification. Additionally, the heater can not only be made in 2D but also in 3D, allowing the customization and fabrication of heaters with complex shapes. We believe that the proposed heater fabrication technique can be utilized as a rapid prototyping technique and help facilitate the development of POCT devices and also positively contribute to the rapidly expanding field of molecular diagnostic research.

## Data Availability

The data presented in this study are available on request from the corresponding author.
